# The WACDT, a modern vigilance task for network defense

**DOI:** 10.3389/fnrgo.2023.1215497

**Published:** 2023-11-21

**Authors:** Oliver A. Guidetti, Craig P. Speelman, Peter Bouhlas

**Affiliations:** ^1^Edith Cowan University, Joondalup, WA, Australia; ^2^Western Australian Department of the Premier and Cabinet, Perth, WA, Australia; ^3^The Cyber Security Research Cooperative, Joondalup, WA, Australia

**Keywords:** cyber security, vigilance, sustained attention, command and control, simulation

## Abstract

Vigilance decrement refers to a psychophysiological decline in the capacity to sustain attention to monotonous tasks after prolonged periods. A plethora of experimental tasks exist for researchers to study vigilance decrement in classic domains such as driving and air traffic control and baggage security; however, the only cyber vigilance tasks reported in the research literature exist in the possession of the United States Air Force (USAF). Moreover, existent cyber vigilance tasks have not kept up with advances in real-world cyber security and consequently no longer accurately reflect the cognitive load associated with modern network defense. The Western Australian Cyber Defense Task (WACDT) was designed, engineered, and validated. Elements of network defense command-and-control consoles that influence the trajectory of vigilance can be adjusted within the WACDT. These elements included cognitive load, event rate, signal salience and workload transitions. Two forms of the WACDT were tested. In static trials, each element was adjusted to its maximum level of processing difficulty. In dynamic trials, these elements were set to increase from their minimum to their maximum values. Vigilance performance in static trials was shown to improve over time. In contrast, dynamic WACDT trials were characterized by vigilance performance declines. The WACDT provides the civilian human factors research community with an up-to-date and validated vigilance task for network defense accessible to civilian researchers.

## Introduction

The natural limitations of the human attentional system are the weakest link in modern cyber defense (Chappelle et al., 2013; Thomason, 2013; Cavelty, 2014). Security Event Information Management Systems (SEIMs) are command and control consoles that network defense analysts are required to sustain vigilant attention (Komlodi et al., 2004; Spathoulas and Katsikas, 2010, 2013; Tyworth et al., 2012; Albayati and Issac, 2015; Newcomb and Hammell, 2016). The United States Air Force Research Laboratory (AFRL) pioneered experimental platforms, known as cyber vigilance tasks, that facilitated studies of sustained attention in network defense analysts (McIntire et al., 2013; Mancuso et al., 2015; Sawyer et al., 2016). Cyber vigilance tasks are designed to emulate the cognitive demands associated with operating a SEIM (McIntire et al., 2013; Mancuso et al., 2015; Sawyer et al., 2016). Existent vigilance tasks, however, are out-dated simulations of the cognitive demands associated with modern network defense and are also preventatively difficult to access by researchers external to the military (McIntire et al., 2013; Mancuso et al., 2014; Sawyer et al., 2016; Guidetti et al., 2023). The Western Australian Cyber Defense Task (WACDT) was developed to fill the need for an updated cyber vigilance task accessible to civilian human factors researchers.

### Research significance

Network defense analysts' vigilance performance has only recently been recognized as a cyber incident risk factor (Chappelle et al., 2013; Mancuso et al., 2014). The capacity of human operators to identify and appropriately defend against virtual threats is bottlenecked by the amount of attention they can sustain for prolonged periods. The WACDT was designed to accurately emulate the cognitive demands associated with SEIM work so it can serve as an experimental platform to study vigilance in network defense. Lessons learned through human factors research conducted with the WACDT could significantly enhance the protective capacity of network defense analysts defending critical cyber infrastructures (Maybury, 2012). For example, increasing reliance on global cyber infrastructures encompasses virtual and physical assets associated with the military, government, central banking, power distribution, and telecommunications (Gordon et al., 2011; Jolley, 2012; Saltzman, 2013; Ormrod, 2014; Hicks, 2015; Skopik et al., 2016; Rajan et al., 2017). The more cyber infrastructures are relied on, the greater the impact of their compromise (Ben-Asher and Gonzalez, 2015; Goutam, 2015). Since the human operator is a bottleneck to the security of cyber infrastructures, an updated experimental platform to study vigilance in network defense is required to address the weakest link in the cyber security chain (Maybury, 2012; Thomason, 2013; Cavelty, 2014). Civilian researchers could, therefore leverage the WACDT to study the human factor bottlenecking cyber infrastructure security (Wall and Williams, 2013).

## Cyber vigilance tasks

### Resource control theory

Defending networks from malicious attacks requires that analysts sustain attention to complex task-relevant processes (Reinerman-Jones et al., 2010; Hancock, 2013). The protective capability of network analysts is determined partly by their capacity to sustain attention to cyber-attacks presented in SEIMs (Jajodia et al., 2011). However, sustaining attention to SEIM alerts is fundamentally an energetically draining experience for analysts. Thomson et al. (2015) theory of resource control can be used to understand sustained attention performance in operational contexts like cyber security. Over time, executive resources allocated to network defense processes decrease, and mistakes begin to snowball (D'Amico et al., 2005; Sarter et al., 2006; Chappelle et al., 2013; Gartenberg et al., 2015; Sawyer et al., 2016; Erola et al., 2017). Lapses in analysts' attention to SEIM alerts due to vigilance decrement can have disastrous effects on network security and severely compromise the integrity of critical cyber infrastructures (Maybury, 2012; Thomason, 2013; Cavelty, 2014). The cyber vigilance task put forward in this study provides an experimental platform by which to probe the attentional capacity of network defense analysts.

### Development challenges

Guidetti et al. (2023) identified cyber-cognitive elements of software design and three central challenges in creating a cyber vigilance task. Firstly, civilian researchers cannot easily access existent vigilance tasks developed within a military context (Paul, 2014; Gutzwiller et al., 2015). The WACDT was therefore developed by civilians to expand cyber vigilance research beyond the military. Secondly, existent cyber vigilance tasks could be presented on a single computer monitor (McIntire et al., 2013; Mancuso et al., 2014; Sawyer et al., 2016; Guidetti et al., 2023). However, modern network defense is too complex a role to perform on a single computer monitor (D'Amico et al., 2016; Axon et al., 2018). The volume and complexity of network defense dashboards often force analysts to divide their attentional resources across two, three, or more computer monitors to interact with the virtual threat landscape (Knott et al., 2013; D'Amico et al., 2016; Axon et al., 2018). The WACDT is a more accurate simulation of the cognitive demands associated with modern network defense, as analysts must sustain attention across three computer monitors, not just one (Knott et al., 2013; D'Amico et al., 2016; Axon et al., 2018).

The final challenge overcome by the WACDT is that SEIM consoles are not designed according to a typical operating design (Reinerman-Jones et al., 2010; Guidetti et al., 2023). SEIM consoles lack a standardized design because each is built according to context-specific cyber security needs (Work, 2020). Hence, designing a modern cyber vigilance task based on any existing SEIM console was impossible, as industry-wide design standards do not characterize these. The WACDT, therefore, had to be designed according to elements of SEIM software design that influence human vigilance performance. For example, Parasuraman (1979, 1985) identified three essential task parameters that can lead to vigilance decrement on sustained attention tasks: cognitive load, event rate, and signal salience. Cognitive load refers to the volume, complexity, and diversity of information that must be retained in working memory while critical signals are appraised (Guidetti et al., 2023). In network defense, critical signals of cyber threats are the alerts visually displayed to analysts on SEIM consoles (McIntire et al., 2013; Mancuso et al., 2015). In cyber security, background event rate refers to how frequently a SEIM presents new information to an analyst surrounding non-threatening network activity (McIntire et al., 2013; Sawyer et al., 2014; Mancuso et al., 2015). Finally, signal salience refers to the modality and clarity by which a SEIM presents malicious network activity to the analyst for their appraisal consoles (McIntire et al., 2013; Mancuso et al., 2015).

Cognitive load, signal salience, and event rate are task features that influence vigilance performance (Grier et al., 2003; Oken et al., 2006; McIntire et al., 2011, 2013; Knott et al., 2013; Sawyer et al., 2014, 2016; Warm et al., 2015, 2018; Neigel et al., 2020). For example, during traditional sustained attention tasks, signal salience, and event rate are directly and inversely related to vigilance performance (Warm et al., 2015, 2018). Sawyer et al. (2014, 2016) likewise demonstrated that cyber vigilance task performance is directly related to signal salience and inversely to event rate. Similarly, the cognitive load associated with network defense has also been associated with vigilance decrement on both traditional and cyber-specific sustained attention tasks (Grier et al., 2003; Oken et al., 2006; McIntire et al., 2011, 2013; Knott et al., 2013; Neigel et al., 2020). Hence, Parasuraman (1979, 1985) parameters were derived from studies of earlier vigilance tasks than those built for network defense. However, Guidetti et al. (2023) review suggested cognitive load, background event rate, and signal salience are also characteristics of SEIM tasks that influence network defense analysts' vigilance performance capacity. Therefore, even though SEIM designs vary immensely across the cyber defense industry, these cyber-cognitive elements of software design that influence analysts' vigilance performance are common across vigilance tasks in general (Silva et al., 2014; Gutzwiller et al., 2015; Vieane et al., 2016).

### Cyber-cognitive elements of SEIMs

The WACDT was designed based on the cyber-cognitive elements of SEIM consoles that influence vigilance decrement in network defense analysts that Guidetti et al. (2023) reviewed. In addition to workload transitions, these encompassed Parasuraman (1979, 1985) original parameters: cognitive load, event rate, and signal salience (Guidetti et al., 2023).

#### Sensitivity to cognitive load

Most of the brain's cognitive and executive functions are superordinate cognitive processes that facilitate planning, problem-solving, response selection, attention regulation and control (Topçuoglu et al., 2009; Harden et al., 2020). The executive functions required to sustain attention depend on task-specific information processing demands. Vigilance performance declines according to task-specific cognitive workload demands (Wickens, 1980, 2002, 2008; Wickens et al., 1985, 2015; See et al., 1995). The behavioral manifestation of vigilance decrement varies according to the cognitive workload associated with sustained discrimination of critical task targets (Guidetti et al., 2023). For example, cyber vigilance problems require the commitment of multiple executive resources that are much greater than those required by classic vigilance domains, such as nuclear plant monitoring, baggage security and air traffic control (Wickens et al., 1997; Hancock and Hart, 2002; Chappelle et al., 2013; Gartenberg et al., 2015; Reinerman-Jones et al., 2016). Typically, this is ascribed to several challenging aspects surrounding the data, which analysts must process to distinguish between malicious and benign SEIM alerts (D'Amico et al., 2005). Challenges include data volume, diversity, and specificity of virtual threat information that must be continuously processed from SEIM consoles (D'Amico et al., 2005; McIntire et al., 2013; Mancuso et al., 2015). Gradual reductions in vigilance task performance cannot be called “vigilance decrement” unless their behavioral presentation changes under different levels of cognitive load (Parasuraman, 1979, 1985). Hence, cognitive load sensitivity is a fundamental criterion by which to design and validate new vigilance tasks (Parasuraman, 1979, 1985).

#### Sensitivity to background event rate

In addition to cognitive load, the rate at which analysts must process new information can amplify performance losses associated with cyber vigilance decrement (Richter et al., 1981; McIntire et al., 2014; Mancuso et al., 2015; Sawyer et al., 2016). This interaction between cyber vigilance decrement and the frequency of background information presentation is known as *The Event Rate Effect* (Richter et al., 1981). For example, accelerating the rate at which information is processed during a cyber incident response exercise will also cause analysts to accelerate the rate at which energy is used up (Thomson et al., 2015). This is analogous to the fuel a car uses when at high vs. low speed. If the driver demands high speed, the fuel used within the car to sustain that activity will not last for the same distance as if the driver demanded a lower speed. In this analogy, the driver represents the vigilance task operator, the speed represents the task's event rate, and the car's fuel represents the executive resources required to sustain performance. Thus, if a vigilance task presents background events relatively quickly, this will accelerate the depletion of the neuronal fuel reserves required to sustain performance. The rate at which background information is presented to analysts over their SEIM contributes to the performance deficits known as cyber vigilance decrement (Mancuso et al., 2015; Sawyer et al., 2016). Cyber vigilance tasks' validity relies on demonstrating a relationship between sustained attention performance reductions and event rate (Parasuraman, 1979, 1985).

#### Sensitivity to signal salience

Some information in an alert will signal a threat, and some will signal a non-threat to the analyst (Heeger, 1997; Sawyer et al., 2014). Analysts must sustain control of their executive functions to weigh both sources of information against each other in assessing the degree of threat presented within the alert (Bridges, 2011; Thomson et al., 2015). However, no SEIM alert is considered in isolation (Alserhani et al., 2010). Network defense analysts must consider alerts relative to the wider virtual threat landscape, communicated through every other alert in the SEIM (Heeger, 1997; Alserhani et al., 2010; Bridges, 2011). This means the analyst must process a second noise level before deciding on a threat designation for any given alert. This second noise level refers to the analyst's contextual knowledge of the wider virtual threat landscape their SEIM presents (Heeger, 1997; Alserhani et al., 2010; Bridges, 2011). That is, the more noise in an SEIM, the more information analysts must process in order to draw a judgment. This translates to an increase in neurological resources used to sustain the executive functions required by that process. However, the more conspicuous the threat component of an alert's information is to an analyst, the less this additional noise decays their performance. If threat salient information is more perceptible, fewer resources are necessary to delineate this against the noisy backdrop of non-critical SEIM alarms.

#### Sensitivity to workload transitions

Workload transitions refer to changes in the level of cognitive load required to perform a task (McKendrick and Harwood, 2019). For example, air traffic controllers must sustain vigilant processing of a variable number of aircraft and non-fixed parameters associated with each, such as speed and trajectory (McKendrick and Harwood, 2019).

Vigilance performance has consistently been demonstrated to be negatively impacted by transitions in task-specific workloads (Krulewitz et al., 1975; Thornton, 1985; Matthews, 1986; Hancock et al., 1995; Cox-Fuenzalida et al., 2004, 2006; Cox-Fuenzalida and Angie, 2005; Cox-Fuenzalida, 2007; Bowers et al., 2014). Transitions in task-specific workload are hence robustly associated in the literature with a cognitive cost that decreases vigilance performance (Krulewitz et al., 1975; Thornton, 1985; Matthews, 1986; Hancock et al., 1995; Cox-Fuenzalida et al., 2004, 2006; Cox-Fuenzalida and Angie, 2005; Cox-Fuenzalida, 2007; Bowers et al., 2014). Workload transitions are also seen in vigilance critical cyber security tasks (Knott et al., 2013).

## The Western Australian cyber defense task

The WACDT is a modern cyber vigilance task developed to accurately simulate the cognitive demands associated with sustained attention tasks in network defense performed with command-and-control consoles. The WACDT presents the user with a simulated network defense dashboard across three computer monitors (referred to as the left, center, and right screen subtasks). The left and right screen subtasks, outlined in the methodology, were designed to explore how signal salience, event rate, and cognitive load impact the user's capacity to sustain vigilant attention to the WACDT. The central screen subtask simulates the cognitive load associated with domain-specific skill use in network defense (Helton and Russell, 2011; McIntire et al., 2013; Mancuso et al., 2015; Vieane et al., 2016).

### Advantages of the WACDT over existent cyber vigilance tasks

The WACDT was designed to overcome several shortcomings of existent vigilance tasks outlined by Guidetti et al. (2023). Firstly, no existent vigilance task simulated the cognitive load associated with domain-specific skill use in network defense (Knott et al., 2013; McIntire et al., 2013; Mancuso et al., 2015; D'Amico et al., 2016; Sawyer et al., 2016; Axon et al., 2018). For example, driving requires sustained vigilant attention to road hazards and a sufficient understanding of how to operate a car (Cox et al., 2000; Satterfield et al., 2019; Fu et al., 2020). Cyber security similarly necessitates two performances: sustaining attention to SEIM consoles and exercising the practical cyber security skills required to triage appropriately and action cyber threats (Naidu and Dharaskar, 2010; Satterfield et al., 2019). Skills central to the cyber security domain are not required to perform cyber vigilance tasks (McIntire et al., 2013; Mancuso et al., 2014; Sawyer et al., 2016; Guidetti et al., 2023). In contrast, performing each of the WACDT's subtasks requires the sustained application of several core cyber security skills. For example, the center and right screens emulate a threat detection task commonly performed in operational network defense (McIntire et al., 2013; Mancuso et al., 2015; Sawyer et al., 2016; BugCrowd., 2020). Anomaly detection is additionally emulated within the right screen subtask, another similarly common task in operational network defense (Keyvanpour et al., 2020).

Secure Socket Layer Blacklist (SSLBL) threat priority rating that the users must memorize before beginning the WACDT (Figures 2, 3). Secure socket layer certificates, or SSLs, are used to detail an organization's identity, location, server name, hostname and domain names (AboutSSL, 2021; Kaspersky, 2021). Secure Socket Layer certificates are used to encrypt communication between clients and servers; however, criminals can misuse them to mask their actions from network defense analysts (AboutSSL, 2021; Kaspersky, 2021). Blacklisted Secure Socket Layer certificates, or SSLBLs, are threats network defense analysts associate with threats discovered in the virtual landscape (AboutSSL, 2021; Kaspersky, 2021). SSLBL certificates are collated by analysts across the globe in large databases, such as BugCrowd. (2020) and the ICE. (2016) project Abuse.ch. SSLBLs are associated with discreet levels of technical severity, which indicate the priority they should be afforded in the work inventory of a network defense analyst (ICE., 2016; BugCrowd., 2020; AboutSSL, 2021; Kaspersky, 2021). Therefore, because analysts use SSLBL ratings to guide anomaly detection in the real world, these are used as threat priorities in the right screen subtask to enhance the WACDT's verisimilitude (BugCrowd., 2020). That is the domain-specific skills required to perform each subtask, therefore, making the WACDT a more realistic simulation of the cognitive workload associated with cyber security than existent cyber vigilance tasks, which do not require any cyber security skill application (Guidetti et al., 2023).

A second shortcoming of existent vigilance tasks is that the demands associated with detecting critical targets are maintained at a fixed or static level (Helton et al., 2004; Chappelle et al., 2013; Knott et al., 2013; Guidetti et al., 2023). However, in the real world, task demands associated with operational network security can dynamically fluctuate rather than remain fixed (Helton et al., 2004). Roles in cyber security often feature frequent transitions in cognitive workload, whereby task-specific processing demands fluctuate dynamically between extremes (Helton et al., 2004; Chappelle et al., 2013; Knott et al., 2013). Neglecting to include workload transitions would have made it harder to generalize any conclusions derived from existent vigilance tasks to cyber vigilance performance beyond the lab (Helton et al., 2004; Chappelle et al., 2013; Knott et al., 2013). Signal salience, event rate and cognitive load were designed as controllable parameters within the WACDT, facilitating the simulation of workload transitions during network defense (Equations 5–8). The WACDT, therefore, provides a more realistic simulation of the cognitive work associated with cyber vigilance performance than any of the existent tasks reviewed by Guidetti et al. (2023), which did not simulate transitions in signal salience, event rate, or cognitive load (McIntire et al., 2013; Mancuso et al., 2014; Sawyer et al., 2016).

**Figure 1:**
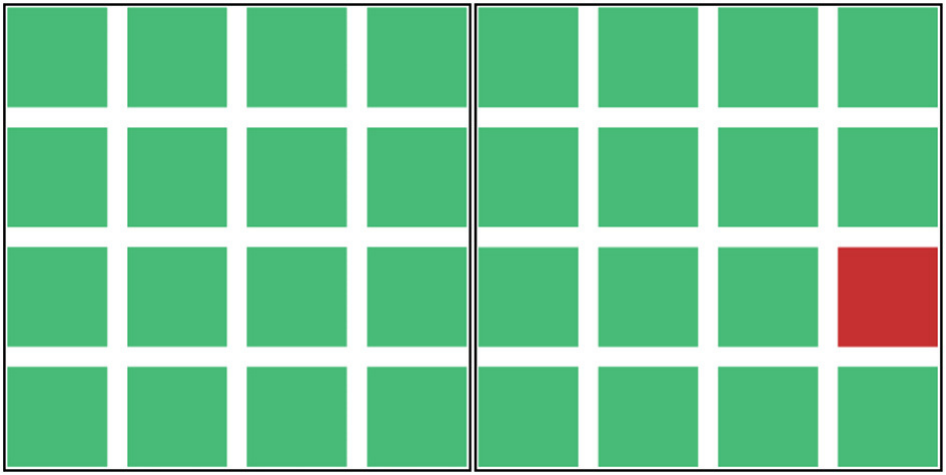
Honeyfile alert system. This figure depicts the honeyfile alert system in the left screen subtask of the WACDT. Icons that begin to flicker from green **(left)** to red **(right)** indicate a potential security breach that the participant must notify a senior analyst about. Participants escalate the alert by activating a macro triggered by simultaneously pressing the “Control” + “D” buttons.

**Figure 2:**
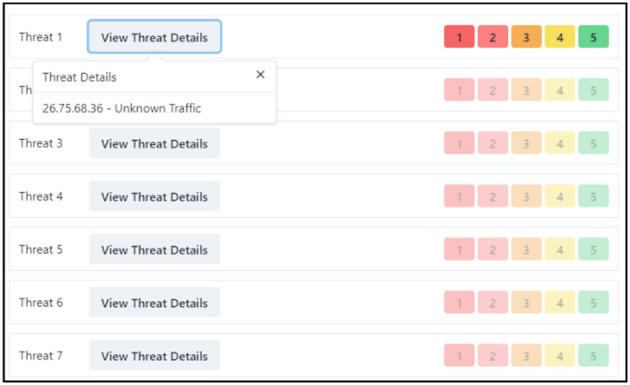
SSLBL alert prioritization and rating on the center screen. This represents the center screen where participants are tasked with assigning correct ratings to SSLBL alerts based on their priority. Each alert discloses the originating IP address. The participant's task includes identifying and rating each alert, with the severity of the alert inversely correlating to its score.

**Figure 3:**
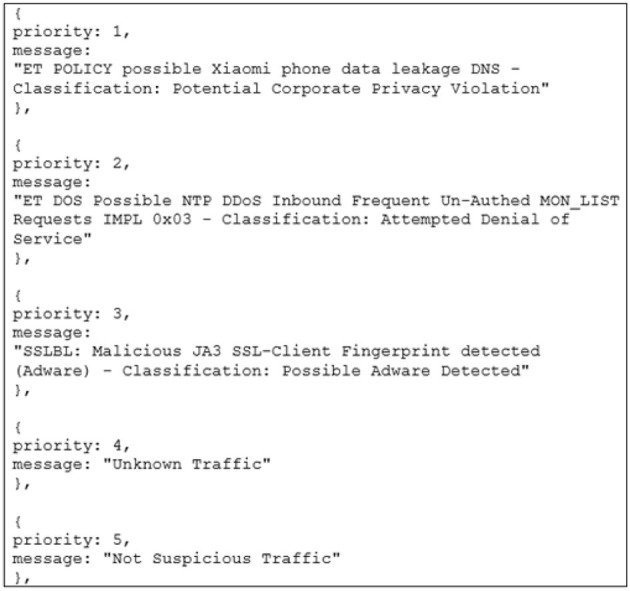
SSLBL alert priority distribution reference sheet. This demonstrates how participants should keep track of the priority for each SSLBL alert. It forms part of the instructions for the center screen task.

**Figure 4:**
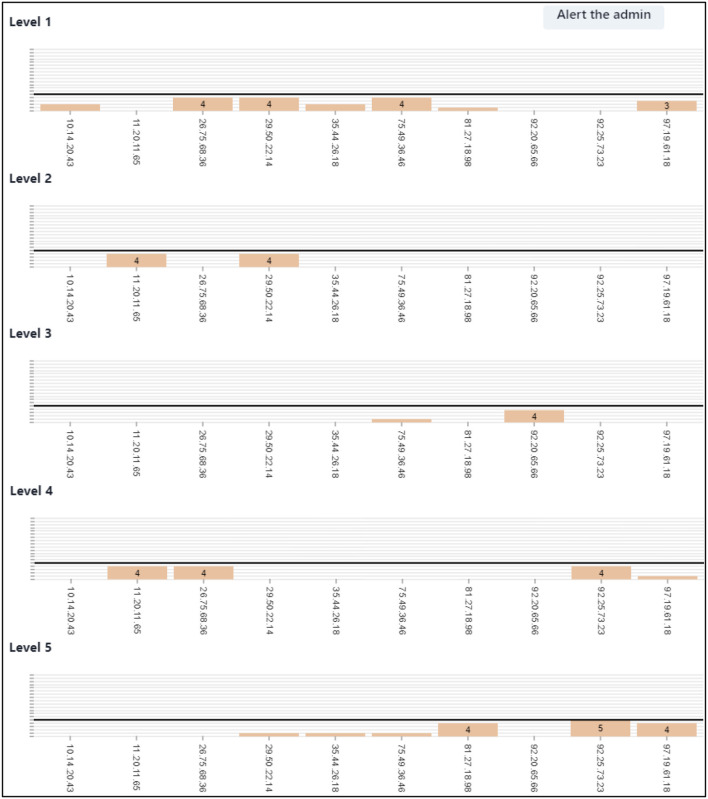
Right screen frequency bar charts and anomaly reporting. This showcases the right screen's evolving frequency bar charts that expand correlating to the rating of alerts. A dark horizontal bar signifies the threshold for an anomalous number of alerts. Participants need to monitor these charts and report any above-threshold activity by clicking “Alert the Admin” and inputting the linked IP address.

**Figure 5:**
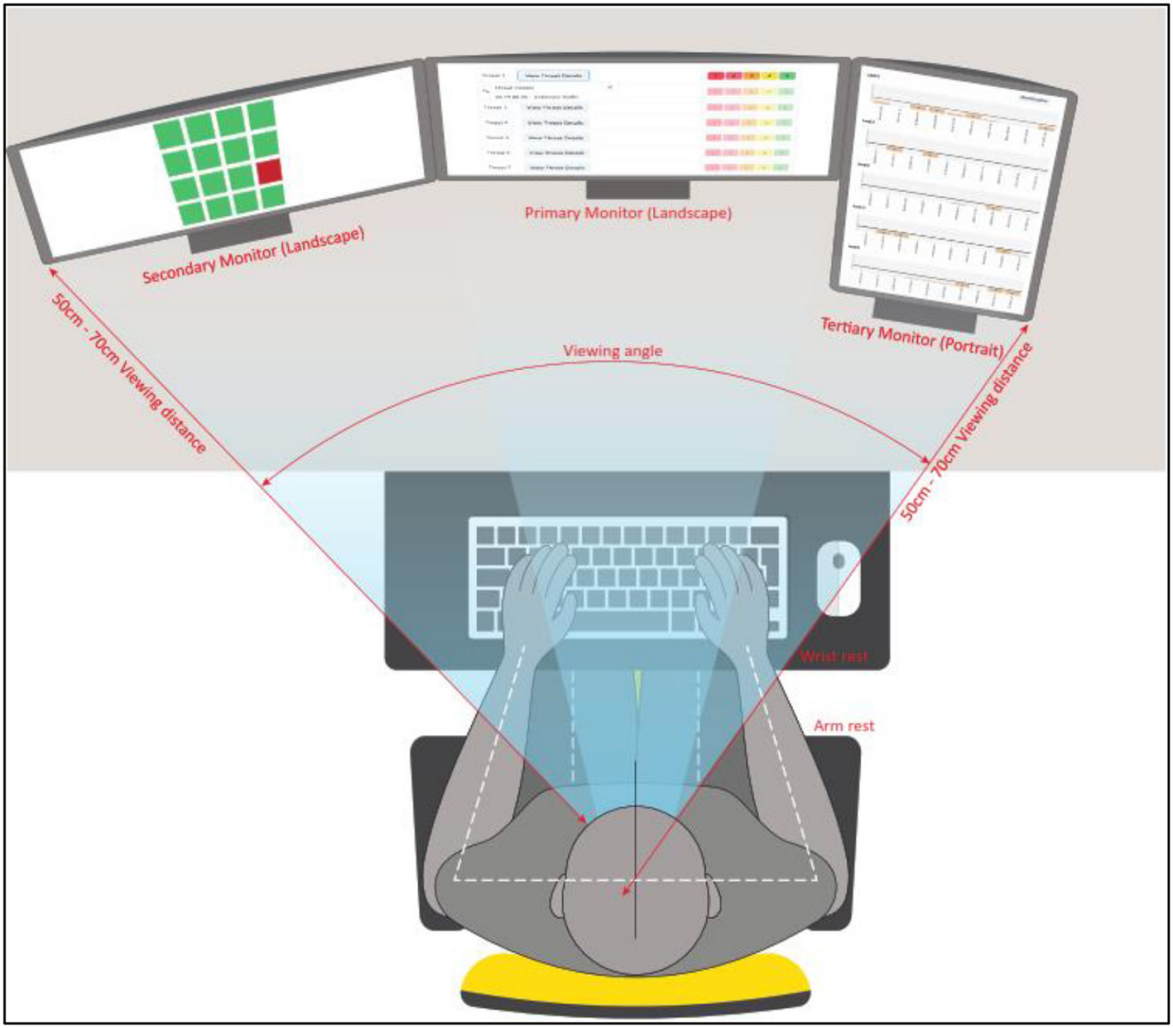
Vertical view of the WACDT setup.

The third shortcoming of existent cyber vigilance tasks considered here is that they require only a single computer monitor. However, modern network defense requires that analysts sustain attention to cyber threats presented across multiple screens displaying relevant network information (D'Amico et al., 2005; Axon et al., 2018). The existent cyber vigilance tasks that Guidetti et al. (2023) reviewed employ a single monitor and hence do not accurately reflect the complexity of modern network defense (McIntire et al., 2013; Mancuso et al., 2014; Sawyer et al., 2016). The WACDT was, therefore, designed to present virtual threat data across three computer monitors (Figures 1–5).

## WACDT validation

### Research design

The validity of the WACDT could not be explored by comparing vigilance performance on it with that observed on existent cyber vigilance tasks, inaccessible to civilian researchers (Guidetti et al., 2023). However, Parasuraman (1979, 1985) suggested that valid vigilance tasks were characterized by declines in sustained attention with time on tasks that depend on the salience of critical signals, event rate, and cognitive load. Parasuraman's parameters provided an alternative method of validating the WACDT.

The validity of the WACDT was explored within two hypotheses. Firstly, if the WACDT is a valid vigilance task, then performance will decline over time. Moreover, Parasuraman suggested that signal salience, event rate and cognitive load impact performance declines in a valid vigilance task. The workload associated with Parasuraman's parameters can be controlled within the WACDT's internal settings (Equations 6–8). The impact of signal salience, event rate and cognitive load on vigilant WACDT performance was explored by testing a dynamic and static task version. In the dynamic WACDT, the cognitive workload was parameterised to dynamically increase in difficulty with time-on-task. In the static version of the WACDT, the cognitive workload was parameterised as static values set to the most challenging level of processing. Dynamic WACDT trials are expected to be harder to perform than the static version, as the former forces users to adapt constantly to an event rate and cognitive load that both increase while signal salience simultaneously decreases all throughout the task. In contrast, there is no additional demand to adapt to changes in event rate, cognitive load, and signal salience during the static WACDT, during which these parameters are held as fixed constants. It was, therefore, secondly hypothesized that if the WACDT is a valid vigilance task, then dynamically changing signal salience, event rate and cognitive load during the dynamic WACDT should lead to greater performance deficits than in the static version where each parameter was kept constant.

### Research objective

The objective behind developing the WACDT was to provide the research field with an accessible, valid vigilance task for network defense. Therefore, this study aims to validate the WACDT so that generalisable conclusions can be derived from cyber security human factors experiments conducted on the platform. The driving question examined by this study was whether the cognitive load, event rate, signal salience, and workload transitions required to correctly detect critical signals in the left, center, and right screen subtasks influenced vigilant performance on the WACDT.

### Participants

After approval was granted from the Edith Cowan University Human Research Ethics Committee (Higher Research Ethics Project Code, 2019-00786), 25 participants were recruited from the Cyber Security Research Cooperative (CSCRC), and the pool of Western Australian Department of Digital Government (WADG) trainee network analysts. This sample size was selected on the basis that Sawyer et al. (2016) recruited 24 participants for their cyber vigilance task study.

The recruited participants had an average age of M_age_ = 35.68 years old with σ_age_ = 11.93 years, slightly younger than the cyber security professional population average of 42 years old (ISC^2^, 2020). Furthermore, women comprise only eleven percent of the global workforce (Poster, 2018; ISC^2^, 2020). Similarly, only twelve percent of the sample were female. Moreover, on average, men and women working in cyber security typically have 6.9 and 5.3 years of experience, respectively (ISC^2^, 2020). Similarly, the men and women who composed this study's sample had an average of 6.4 and 5.0 years of experience working in cyber security, respectively. Therefore, whilst the participants in the sample were younger than the population average, their gender distribution and range of work experience in cyber security reasonably approximated that of the wider population of network defense professionals (ISC^2^, 2020). For convenience, participation took place in the Western Australian Office of Digital Government's offices to minimize disruption to the participants.

### Equipment

**Figure 6:**
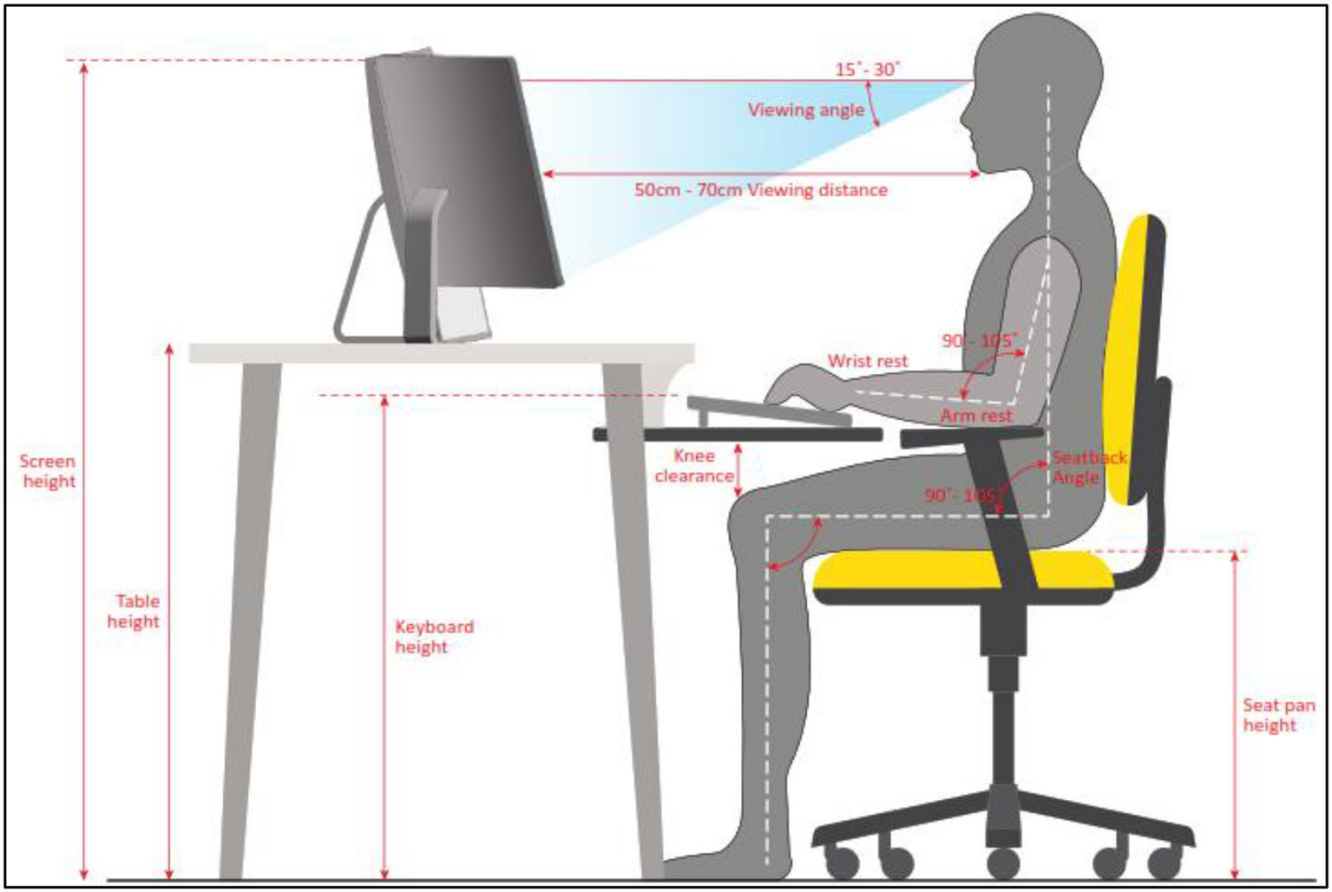
Horizontal view of the WACDT setup.

**Figure 7:**
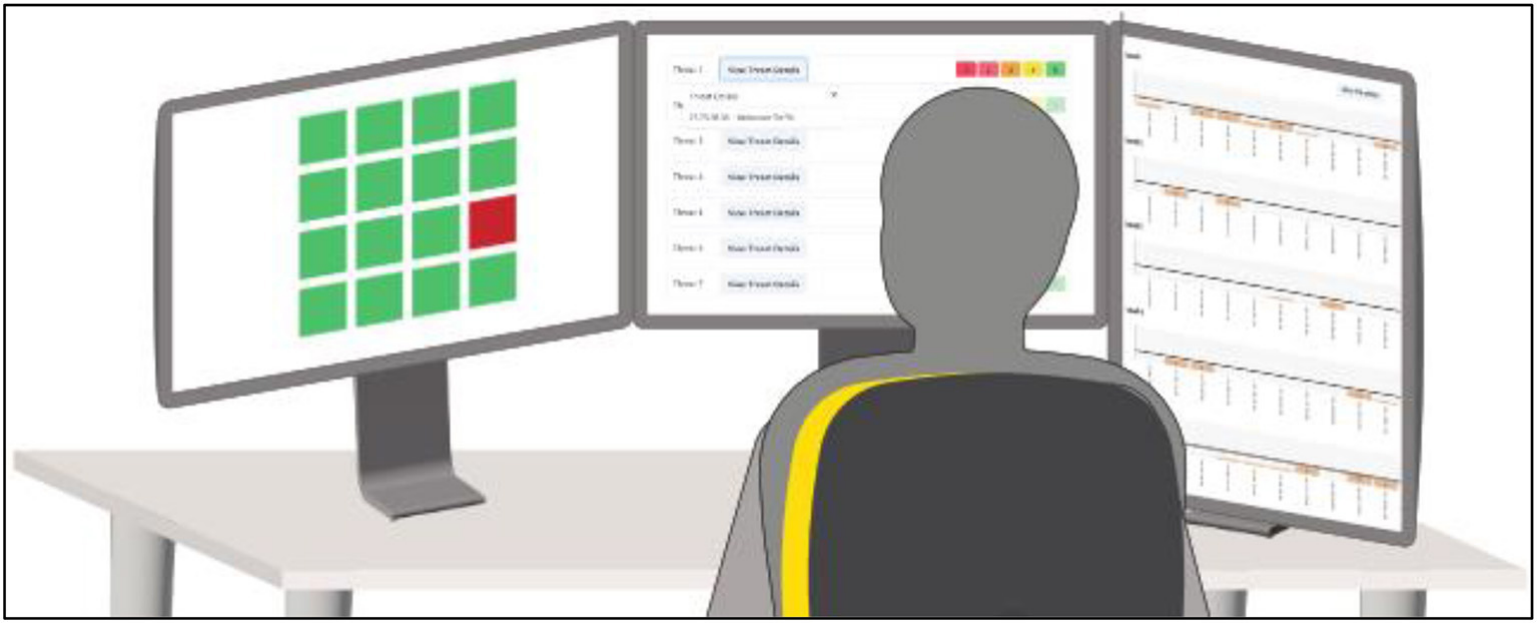
Over the shoulder view of the WACDT setup. This depicts the positioning of each WACDT subtask across three distinct computer displays, as seen by an experimenter over the shoulder of a participant.

Participants completed the WACDT in an isolated room, using a single computer with three computer monitors, a keyboard, and a mouse (Figures 5–7). Each computer monitor was used to run one of the three subtasks of the WACDT that participants completed simultaneously. The left, center, and right screen subtasks of the WACDT outlined here were designed to explore how signal salience, event rate, and cognitive load impact the user's capacity to sustain vigilant attention to virtual threat landscapes.

### WACDT left screen subtask

The purpose of the central screen subtask was to simulate the cognitive load associated with domain-specific skill use in network defense, namely monitoring a system of honeyfiles (Figure 1) (Helton and Russell, 2011; McIntire et al., 2013; Mancuso et al., 2015; Vieane et al., 2016). Network defense analysts use honeyfiles to guard against data theft and unauthorized system access (Whitham, 2016). Honeyfiles are designed to resemble “real” documents that attract data thieves. Network defense analysts are prompted with a security alert any time a honeyfile is interacted with, including being opened, copied, deleted or moved around (Whitham, 2016). Data thieves are susceptible to honeyfile traps primarily because they are indistinguishable from legitimate system files (Tirenin and Faatz, 1999; Yuill et al., 2004; Voris et al., 2013).

Honeyfile alerts were selected as a basis for the left-screen subtask. An array of sixteen square icons is presented to the user on the left screen subtask. These icons illustrate the security status of honey files distributed within a highly sensitive sub-network. Green icons indicate an uncompromised, secure sub-network, and icons that flicker between red and green indicate a honeyfile that has been opened, copied, deleted, moved, or otherwise compromised.

The sixteen icons in the left screen sub-task illustrate a specific honey file's security status within a highly sensitive sub-network that the user is told requires protection. Honey file icons that are un-compromised display a steady color of green. When a honey file is compromised, an alert is triggered that causes the relevant icon to rapidly switch color between green and red, which is referred to as “flickering.” All sixteen icons are in an uncompromised (green) state at the beginning of the WACDT and randomly begin to flicker from red to green increasingly as time progresses on the task. The frequency that icons can be made to flicker between 20 and 48 times per second. The exact placement of honeyfile icons is not crucial to the main task. The reason for displaying 16 icons was to ensure that the operators' attention was distributed across a wide field of view and across enough icons to make the location of a given alert uncertain at any given point in time.

Keyboard macros are recorded keystroke combinations that can trigger software operations (Gunnarson, 1993). Encoding repetitive, monotonous features of a task into a keyboard macro can reduce network defense analysts' steps to command and control a SEIM console (Gillespie, 1986). Network defense is just one of many computer science sub-domains where keyboard macros are commonly used (Kurlander, 1993). A keyboard macro was hence used as the mechanism to be actioned when a user identifies a critical alert on the WACDT's left screen subtask.

Specifically, the user is told to press “Control” and “D” as soon as they notice a left screen icon flickering between red and green. Simultaneously pressing the “Control” and “D” buttons causes the left screen icon to stop flickering and immediately revert to green. The WACDT registers a left hit when the user presses “Control” and “D” after an icon begins to flicker. If a user performs an action other than “Control” and “D” after an icon begins to flicker, the WACDT registers this as a left miss. A left screen false alarm is registered if the user presses “Control” and “D” when no icon is flickering.

### WACDT central screen subtask

The center screen threat intelligence subtask demands domain-specific skills from the participating network analyst. The center screen presents the participant with a queue of common operational network threat alerts (BugCrowd., 2020) (Figures 2, 3). Center screen alerts encompass an originating IP address and an SSLBL threat priority rating that users commit to memory before beginning the WACDT (Figures 2, 3). The WACDT registers a hit on the center screen if the user's threat score matches the alert's SSLBL rating. If an alert is given a lower threat score than its SSLBL rating, the WACDT registers a false alarm. A miss is registered when an alert is given a higher threat score than its SSLBL rating. Basing center screen alerts on SSLBL scores improves the WACDT's concordance with the kind of information network defense analysts process in the real world. For example, more severe security breaches have lower scores, as during an incident response, these would be closer to a top priority.

### WACDT right screen subtask

The center and right subtasks are designed to simulate the cognitive load associated with anomaly detection, a prevalent domain-specific skill used in network defense (McIntire et al., 2013; Mancuso et al., 2015; Sawyer et al., 2016). Anomaly detection is a SEIM task that involves the identification of non-conformal patterns and features, also known as outliers or contaminants, in subsets of network traffic data (Chandola et al., 2010; Alabadi and Celik, 2020). Government, military and private businesses derive critical and actionable insights from anomalies detected in data (Chandola et al., 2010). For example, financial anomalies are a signature of identity or monetary theft, anomalies in MRI data can indicate cancer, and anomalous spaceship telemetry could cause massive loss of life (Aleskerov et al., 1997; Spence et al., 2001; Fujimaki et al., 2005; Kumar, 2005). Anomalous patterns of network traffic are signals of malicious cybercrime (Kumar, 2005). For example, data exfiltration attacks target repositories of sensitive information, which can be leaked publicly, sold to an unauthorized external party, or held for ransom (Kumar, 2005). Network defense analysts must monitor inbound and outbound traffic for signatures that suggest a malicious actor has deployed a data exfiltration attack (Kumar, 2005).

Anomaly detection has become an increasingly critical skill for network defense analysts (Kumar, 2005). The right screen subtask was therefore designed to simulate SEIM anomaly detection by requiring users to identify non-conformal patterns and features in the network traffic initially presented on the center screen (Chandola et al., 2010; Alabadi and Celik, 2020; Keyvanpour et al., 2020).

What constitutes an anomalous volume of alerts that requires the attention of a network defense analyst differs across organizations, network environments, and threat types (Bhatt et al., 2014). Admin alert thresholds are pre-defined values that signify an anomalous number of alerts within the WACDT (Coviello and Mariniello, 2010). The SSLBL score of an alert is directly related to the admin alert threshold it is associated with. For example, the SSLBL score for a corporate privacy violation is 1 and has an admin alert threshold of 3, whereas unknown traffic has an SSLBL score of 4 and an admin alert threshold of 9 (BugCrowd., 2020). An admin alert threshold has hence been assigned to each of the five types of alerts integrated into the WACDT (Table 1). The right screen contains five frequency charts, one for each of the types of alerts integrated into the WACDT.

**Table 1:** Threat alert threshold values.

**Threat priority rating**	**Admin alert threshold value**
1	3
2	5
3	7
4	9
5	11

The IP address of each alert, processed by the user in the center screen, is presented along the horizontal axis of each right screen chart (Figure 4). The vertical axes of each chart present a count of the number of alerts detected from each of these originating IP addresses. As the user rates the SSLBL score of each center screen alert, their entries cause the columns within the frequency charts on the right screen to grow.

Users are told that they must monitor the right screen for chart columns that reach the anomaly threshold that is indicated by a dark horizontal bar (Figure 4). Users are required to then click the “Alert the admin” button when they see this, and enter the IP address associated with anomalous chart elements. When the correct IP address for an anomaly is entered, this causes its associated column chart element to reset to zero. This variation in anomaly thresholds helps minimize harm in real-world network defense, as it allows network defense analysts to prioritize response resources and actions more efficiently (Vilendečić et al., 2017).

A right screen hit is hence registered by the WACDT when the user correctly enters the IP address of a threshold-breaking bar chart element. A miss is registered if a right screen bar chart breaches the horizontal threshold line and the user does anything other than enter that bar chart's IP address into the admin alert text box. This includes reacting to left screen signals or continuing to work through the center screen work queue. A right screen false alarm is registered when the user enters an incorrect IP address into the “Alert the admin” window.

### WACDT positioning

A trio of computer monitors were used to complete the WACDT, which was completed on an ergonomically arranged computer setup. The WACDT sub-tasks were positioned on the left, center, and right screens. These screens are referred to as the secondary, primary, and tertiary monitors, respectively. The center screen task is presented on the primary monitor in landscape orientation and directly in front of the user. The left screen sub-task is displayed on the secondary monitor situated to the left of the primary monitor. The right screen sub-task is displayed on the tertiary monitor situated to the right of the primary monitor. Each display is 59.9 cm along the diagonal, with a width of 50.8 cm and a height of 31.8 cm. Size twelve font was used in all textual elements of the WACDT, including information in the center screen alerts and the IP addresses in the right screen sub-task.

#### Primary monitor

The center screen sub-task is displayed on the primary monitor, which is positioned directly in front of the user, who sits approximately an arm's length away. The top of the primary monitor is aligned with the users' eye level to reduce neck strain.

#### Secondary monitor

The left screen sub-task is displayed on the secondary monitor, and this is positioned directly to the left of the primary monitor to avoid a visual gap. The secondary monitor is positioned at the same height as the primary monitor and aligns with the user's eye level.

#### Tertiary monitor

The right screen sub-task is displayed on the tertiary monitor, which is in a portrait orientation and is positioned at the same height and to the right of the primary monitor.

#### Monitor angles

The primary monitor is positioned directly in front of the user. The secondary and tertiary monitors were angled toward the user to minimize head movement. More specifically, the secondary monitor, which displays the left screen sub-task, is angled toward the user by approximately 15 to 30 degrees to minimize head movement while performing the task. Similarly, the tertiary monitor was angled toward the user by around 15 to 30 degrees. This arrangement gave the primary, secondary and tertiary monitors a gentle curve, making it easier for the user to glance from one screen to the next.

#### Keyboard and mouse placement

The keyboard and mouse were positioned so their elbows rested at a comfortable angle, approximately 90 degrees, to ensure straight wrists that are not bent upwards or downwards while typing or moving the mouse.

#### Lighting

The experiment was undertaken in a windowless room under standard office lighting to minimize glare on the computer monitors.

### Behavioral parameters tracked by the WACDT

The concept of hits, misses, and false alarms from signal detection theory were adapted to parameterise performance on the WACDT. These terms have only been conceptually borrowed from Signal Detection Theory to describe how well users respond to the various signals presented across the left, center and right screen subtasks of the WACDT.

#### Left screen sub-task performance metrics: hits, misses, and false alarms

- Hit: “Control” + “D” after a left screen icon begins to flicker.
- Miss: Performing any WACDT action other than pressing “Control” + “D” when an icon flickers.
- False Alarm: Pressing “Control” + “D” when no icon is flickering.

#### Center screen sub-task performance metrics: hits, misses, and false alarms

- Hit: Correctly rating the threat level of an alert.
- Miss: Underestimating the threat level of an alert.
- False Alarm: Overestimating the threat level of an alert.

#### Right screen sub-task performance metrics: hits, misses, and false alarms

- Hit: Entering the correct IP address when a bar chart column crosses the threshold.
- Miss: Doing anything other than entering the correct IP after a column crosses the threshold.
- False Alarm: Entering an incorrect IP address when no column has crossed the threshold.

#### Notes on terminology

- Miss vs. Non-Response: A “miss” involves some action but the wrong one, while a “non-response” involves no action at all.
- Miss vs. Error of Commission: A “miss” is a specific incorrect action within the WACDT, while an “error of commission” could be any incorrect action.
- False Alarm vs. Error of Commission: A “false alarm” is a specific action taken in the absence of a stimulus, while an “error of commission” is an incorrect action taken in the presence of a stimulus.

The WACDT tracks and outputs the minute-by-minute total number of hits, misses, and false alarms to critical signals across each screen subtask. That is, $\frac{dH}{dt}$, $\frac{dM}{dt}$, and $\frac{dF}{dt}$ are taken as the total count of all hits, misses, and false alarms recorded per minute on the WACDT. More specifically, the WACDT tracks and records the number of hits, misses, and false alarms made per minute on the left, center, and right screen subtask, $\frac{dH_{Left}}{dt}$, $\frac{dM_{Left}}{dt}$, $\frac{dF_{Left}}{dt}$, $\frac{dH_{Centre}}{dt}$, $\frac{dM_{Centre}}{dt}$, $\frac{dF_{Centre}}{dt}$, $\frac{dH_{Right}}{dt}$, $\frac{dM_{Right}}{dt}$, and $\frac{dF_{Right}}{dt}$. The variable, “t” is a continuous variable taken to represent time, ranging from 0 min to 60 min on the WACDT. “dt” denotes an infinitesimally small change in of time. The WACDT tracks and records the number of hits, misses, and false alarms made per minute on the left, center, and right screen subtask, denoted by dH_Left_, dM_Left_, dF_Left_, dH_Centre_, dM_Centre_, dF_Centre_, dH_Right_, dM_Right_, and dF_Right_ (Equations 1–4). As a fraction, $\frac{dH_{Left}}{dt}$, $\frac{dM_{Left}}{dt}$, $\frac{dF_{Left}}{dt}$, $\frac{dH_{Center}}{dt}$, $\frac{dM_{Center}}{dt}$, $\frac{dF_{Center}}{dt}$, $\frac{dH_{Right}}{dt}$, $\frac{dM_{Right}}{dt}$, and $\frac{dF_{Right}}{dt}$ reflect differential changes in the rate that hits, misses, and false alarms are made on the left, center, and right subtasks. Vigilance performance on the WACDT is then parameterised as the 2-min bucket average correct detection percentage on the left, center, and right screen subtasks, as well as across the total WACDT and is denoted by the component $100\int \begin{array}{c} t+2\\ t \end{array}$ (Equations 1–4). Recording hits, misses, and false alarms in this way allows performance on the WACDT to be explored as a whole or at the level of the individual subtask.

Left Screen Vigilance Performance : = *L*(*t*),


(1) $$L(t)=\frac{100\int_{t}^{t+2}\left\{\frac{dH_{Left}}{dt}\right\}}{\int_{t}^{t+2}\left\{\frac{dH_{Left}}{dt}+\frac{dM_{Left}}{dt}+\frac{dF_{Left}}{dt}\;\right\}}$$

Center Screen Vigilance Performance : = *C*(*t*),


(2) $$C\left(t\right)=\frac{100\int_{t}^{t+2}\left\{\frac{dH_{Centre}}{dt}\right\}}{\int_{t}^{t+2}\left\{\frac{dH_{Centre}}{dt}+\frac{dM_{Centre}}{dt}+\frac{dF_{Centre}}{dt}\right\}}$$

Right Screen Vigilance Performance : = *R*(*t*),


(3) $$R(t)=\frac{100\int_{t}^{t+2}\left\{\frac{dH_{Right}}{dt}\right\}}{\int_{t}^{t+2}\left\{\frac{dH_{Right}}{dt}+\frac{dM_{Right}}{dt}+\frac{dF_{Right}}{dt}\right\}}$$

Total WACDT Vigilance Performance : = *T*(*t*),


(4) $$T(t)=\frac{100\left(\int_{t}^{t+2}\left\{\frac{dH_{L}}{dt}\right\}+\int_{t}^{t+2}\left\{\frac{dH_{Centre}}{dt}\right\}+\int_{t}^{t+2}\left\{\frac{dH_{Right}}{dt}\right\}\right)}{\int_{t}^{t+2}\left\{\frac{dH_{Left}}{dt}+\frac{dM_{Left}}{dt}+\frac{dF_{Left}}{dt}\right\}+\int_{t}^{t+2}\left\{\frac{dH_{Centre}}{dt}+\frac{dM_{Centre}}{dt}+\frac{dF_{Centre}}{dt}\right\}+\int_{t}^{t+2}\left\{\frac{dH_{Right}}{dt}+\frac{dM_{Right}}{dt}+\frac{dF_{Right}}{dt}\right\}}$$

### Parameterizing the WACDT's neurocognitive workload factors

The cognitive demands associated with real-world network defense are more than static values and can dynamically change within a short window of time (Vieane et al., 2016). For example, a prevalence denial attack involves flooding a network defense analysts' system with a high number of low-level threats to mask the presence of a more malicious attack (Vieane et al., 2016). Signal salience, event rate, and cognitive load in the WACDT were hence engineered to vary between natural human processing boundaries (Glassman et al., 1998; Shady et al., 2004; Tse et al., 2004; Herbst et al., 2013; Alais et al., 2016; Sawyer et al., 2016). This feature distinguished the WACDT from previous cyber vigilance tasks, which explored cyber vigilance performance under static cognitive demand conditions (McIntire et al., 2013; Mancuso et al., 2015; Sawyer et al., 2016).

Each parameterising expression for signal salience, event rate and cognitive load adopt the use of “t” and “dt” to respectively reflect time in minutes, and an infinitesimally small change in time (Equations 5–8). Hence the expression “∀t∈[0,60]” reflects all values of time within the interval of 0 min to 60 min, which is the duration of each WACDT trial.

#### Parameterizing signal salience

The left screen subtask served as the mechanism by which signal salience was controlled. Critical left screen signals manifested as blinking icons that flickered between green and red at a particular frequency (ℱ, Hz). Flickering icons are common operational critical signals used to communicate critical threat salient information to the user (Shady et al., 2004; Tse et al., 2004; Herbst et al., 2013). For example, Alais et al. (2016) demonstrated that attention to flickering signals was optimized at a flicker frequency between 20 Hz and 48 Hz. Beyond 48 Hz, Alais et al. (2016) demonstrated that the probability that a human could detect a flickering icon decreased to chance. Signal salience, S(t), was parameterised according to Alais et al. (2016) perceptual flicker boundaries to linearly increase processing difficulty with time on task. However, by setting the gradient to zero within the WACDT's internal settings, signal salience could also be held as a fixed constant (Equation 5).


(5) $$\begin{array}{l} S(t)\;=\;\{\begin{array}{c} \begin{array}{c} ℱ=\;20Hz\\ ℱ\;=\;48Hz\; \end{array}\begin{array}{c} \;at\;t\;=\;0\;minutes\\ \;at\;t\;=\;60\;minutes \end{array} \end{array}\\ \;\Rightarrow S(t)\;=\frac{7}{15}t+20\;\forall t\in [0,60]. \end{array}$$

#### Parameterizing event rate

The left screen subtask was the mechanism by which the event rate, E(t), was controlled as a linear function of time and spanned between Sawyer et al. (2016) event rate domain of eight to sixteen events per minute. Therefore, the rate that critical left screen signals presented during the WACDT was defined by Equation 6.


(6) $$\begin{array}{l} E(t)\;=\;\{\begin{array}{c} \begin{array}{c} 8\;events\;per\;minute\;\\ 16\;events\;per\;minute\; \end{array}\begin{array}{c} at\;t\;=\;0\;minutes\\ \;at\;t\;=\;60\;minutes \end{array} \end{array}\\ \;\Rightarrow E(t)\;=\frac{2}{15}t\;+8\;\forall t\in [0,60]. \end{array}$$

#### Parameterizing cognitive load

As the user processes center screen alerts, they must remember which right screen chart elements had a count one below the admin alert threshold. These are referred to as near-critical right screen signals. A fictitious artificial intelligence (AI) was built into the WACDT to control the number of near-critical right screen signals presented at any time. The user is told that the AI provides additional column chart elements uncovered elsewhere in the network as they work.

The right screen AI mechanism is hence used to control cognitive load as a linear function of time, bounded by natural human processing limitations associated with working memory. Miller (1956) demonstrated that human working memory capacity is generally limited to 7 ± 2 elements. However, cognitive load is not bounded between one and nine because the user already retains five items in working memory in performing the center screen subtask. Instead, cognitive load is bounded between one and four elements that are added to randomly selected column chart elements throughout the WACDT (Equation 7).


(7) $$\begin{array}{l} C(t)=\;\left\{\begin{array}{c} \begin{array}{c} Near-critical\;right\;screen\;signals=\;1\;t\;=\;0 \end{array}\\ \begin{array}{c} Near-critical\;right\;screen\;signals\;=\;4\;t\;=\;60 \end{array} \end{array}\right\}\\ \;\Rightarrow C(t)\;=\;\frac{1}{20}t\;+\;1\;\forall t\in \left[0,60\right]. \end{array}$$

The components of the vector function describe the neurocognitive workload associated with the WACDT, $\tilde{w}$ (Equation 8). These components of the WACDT control the implementation of Parasuraman (1979, 1985) parameters as functions of time-on-task to capture the dynamic cognitive workloads required in operational network defense.


(8) $$\begin{array}{l} \tilde{w}\;=\;\left[\begin{array}{c} S(t)\\ E(t)\\ C(t) \end{array}\right]\;\forall t\in [0,60]\\ \;\Rightarrow \tilde{w}\;=\;\left[\begin{array}{c} \frac{7}{15}t+20\;\\ \frac{2}{15}t\;+8\\ \;\frac{2}{15}t\;+\;1 \end{array}\right]\\ \;\Rightarrow \tilde{w}\;=\;\left[\begin{array}{c} \frac{7}{15}\;\\ \frac{2}{15}\;\\ \frac{2}{15} \end{array}\right]t+\left[\begin{array}{c} 20\;\\ 8\\ \;1 \end{array}\right]\;\forall t\in [\;0,60]. \end{array}$$

## Methodology

### Procedure

Each subtask was run concurrently during each trial. This meant that each participant completed two trials of the WACDT, one static, and one dynamic. The timing of each trial was the same across the static and dynamic conditions, 60 min. The timing of each WACDT trial was determined by Guidetti et al. (2023) review of existing cyber vigilance tasks presented by McIntire et al. (2013), Mancuso et al. (2015), and Sawyer et al. (2016). In these prior studies, the duration of each cyber vigilance task was limited to 50, 50, and 40 min, respectively. Since time on task is a factor that influences vigilance decrement, it was decided to round up the duration of each WACDT trial to a full hour (Ziino and Ponsford, 2006). By extending the WACDT's trial duration beyond that Guidetti et al. (2023) reported in previous studies, allows for a more nuanced observation of changes in cyber vigilance performance. This longer time frame provides a more robust examination of participants' performance on the WACDT task. The duration of each WACDT was therefore fixed at 60 min for both the static and dynamic conditions.

Intra-trial learning effects could confound any continuous vigilance performance data analysis recorded during the WACDT (Valcour et al., 2009). Thus, the only randomized component of the study was whether participants performed under static conditions first or if they performed under dynamic conditions first. Stimuli position, text and information were not randomized between the left, center, and right-screen subtasks. In the Western Australian Cyber Defense Task (WACDT), the static and dynamic conditions were controlled by manipulating task workload factors (Equation 8). These factors increased processing difficulty over time for the dynamic version of the WACDT. The WACDT was made with Python, and so the variation between the static and dynamic conditions needed to be controlled by implementing Equations 5–8, within the WACDT's back end. Conversely, the static version of the WACDT featured task workload factors that were held constant at their most difficult processing values, which again were governed by the same equations. What was randomized was the type of trial that individual participants completed. More specifically, balanced randomization was used to manage the risk that learning effects could confound vigilance performance on either the static or dynamic WACDT (Engleman et al., 1998). Participants were allocated to one of two protocols: either they completed the dynamic form of the WACDT first, or their first trial was completed under static conditions.

After providing their informed consent, each of the sub-tasks was explained to participants. The subtasks for the left, center, and right screens were explained to participants in the following manner:

1. The responsible investigator began by addressing the left screen's functionality. The responsible investigator informed participants that if any icon on the left screen flickered between red and green, it signified a compromise in the honeyfile system. Participants needed to notify a senior analyst about the potential breach in such cases by activating a macro, which the responsible investigator explained could be triggered by simultaneously pressing the “Control” and “D” buttons.
2. Moving on to the center and right screens, the responsible investigator clarified that participants must remember the priority of each SSLBL alert (Figure 3). They were then tasked with assigning the correct rating to each alert in the center screen queue (Figure 2).
3. The investigator further explained that every center screen alert includes the originating IP address. The responsible investigator explained that as participants rate alerts, the frequency bar charts on the right screen would expand (Figure 4). The dark horizontal bar on each graph of the right screen represents the threshold for an anomalous number of alerts.
4. The responsible investigator lastly guided participants on how to report anomalies. If they detected alert levels surpassing the threshold, they were to click the “Alert the admin” button and input the IP address associated with the anomalous bar chart into a provided text box (Figure 4). The were then shown the five types of alerts.

The Responsible Investigator demonstrated the WACDT before participants began each trial.

### Ethics

The Responsible Investigator contacted potential participants from the CSCRC and WADG by email after ethical approval to do so had been granted by the Edith Cowan University Human Research Ethics Committee. Information letters were then sent to those who replied to the initial email that described what would be required of them during the study. Participation dates and times were arranged for each network defense analyst who volunteered for the research. It was explained that participants' data would be anonymised, that they retained the right to withdraw from the research at any stage without prejudice, and that they would be remunerated for each completed WACDT with a $50.00 gift card. Participants who decided to withdraw consent would have had their data and any record of their involvement in the project erased. However, no participant withdrew their consent.

## Results

### Data analysis

Vigilance decrement refers to a decline in sustained attention task performance over time (Parasuraman, 1979, 1985; Wickens, 1980, 2002, 2008; Wickens et al., 1985, 2015; See et al., 1995). Moreover, the direction in which vigilance performance changes with time-on-task is known as its “trajectory” (MacLean et al., 2009). For example, a positive trajectory would suggest a net increase in vigilance performance with increasing time spent on the WACDT (MacLean et al., 2009). In contrast, a negative trajectory would suggest vigilance decrement (MacLean et al., 2009). The WACDT keeps a count of the number of hits, misses and false alarms participants make on the left, center, and right screen subtasks, which were then transformed into the percentage of correctly detected alerts in the WACDT (Equations 1–4). Vigilance performance across the total set of sub-tasks, T(t). T(t), L(t), C(t), and R(t) of the WACDT were then computed as the ensemble average of all participants' detection percentages for static, S_avg_, and dynamic D_avg_ trials.

The Kendall (1962) and Sen (1968) slope analyses are non-parametric tests and are more appropriate for exploring the trajectories of vigilance performance on the WACDT for several reasons. This is because Sen's slope and the Mann-Kendall estimates can detect monotonic trends in vigilance performance without an underlying assumption about the data's structure or distribution (Mustapha, 2013). Trends estimated by regression are based on minimizing the difference between observed values and values predicted according to a presupposed function, which means they are less flexible at exploring more complex changes in data. In contrast to regression, trends estimated by Mann-Kendall and Sen's slope analyses are robust against abrupt jumps or heavy tails, which negates the need to pre-process the data (Cao et al., 2011; Chantre et al., 2014). Moreover, the only assumption of Sen's slope and the Mann-Kendall tests is that data are not autocorrelated (Hamed and Rao, 1998; Koutsoyiannis, 2003). However, time series data derived by observation, such as that generated by the WACDT, are commonly autocorrelated (Caloiero et al., 2018). If the assumption of no autocorrelation is violated, the trend significance levels generated by the Sen's slope and Mann-Kendall tests can be underestimated (Hamed and Rao, 1998; Koutsoyiannis, 2003). Hence, despite the advantages of using the Mann-Kendall and Sen's slope analyses to explore changes in WACDT vigilance performance, additional steps were needed to ensure that these analyses were robust against this assumption violation. To this end, a Durbin (1950) autocorrelation test was used to explore autocorrelation within each participant's vigilance performance data. Secondarily to the Durbin-Watson test, each participant's vigilance performance curve was graphed so that the trends estimated by the Mann-Kendall and Sen's slope analyses could be compared with the direction observed through visual inspection of the data. That is, the outputs of each analysis were validated by comparison to a graphical plot of the trend component of each WACDT vigilance performance curve.

T(t), L(t), C(t), and R(t) were formed by the WACDTs' vigilance performance curves (VPCs) for S_avg_ and D_avg_. The validity of the WACDT firstly relied on demonstrating that task performance declines over time. Demonstrating that declines in performance are sensitive to cognitive load, event rate, signal salience, and workload transitions is the second requirement of validating the WACDT (Parasuraman, 1979, 1985; Guidetti et al., 2023). The static and dynamic versions of the WACDT held these parameters as constants or changed accordingly. Firstly, a negative performance trajectory would suggest vigilance decrement and hence satisfy the first requirement in demonstrating the WACDT's validity of the WACDT. In contrast, a positive performance trajectory would not suggest that the WACDT is a valid vigilance task (Parasuraman, 1979, 1985). The second requirement of validating the WACDT would be satisfied by the observation of relative differences in the performance trajectories derived from static and dynamic trials, as this would suggest sustained attention to the task is sensitive to cognitive load, event rate, signal salience, and workload transitions (Parasuraman, 1979, 1985; Guidetti et al., 2023).

### Averaged results

#### Total task vigilance performance

**Figure 8:**
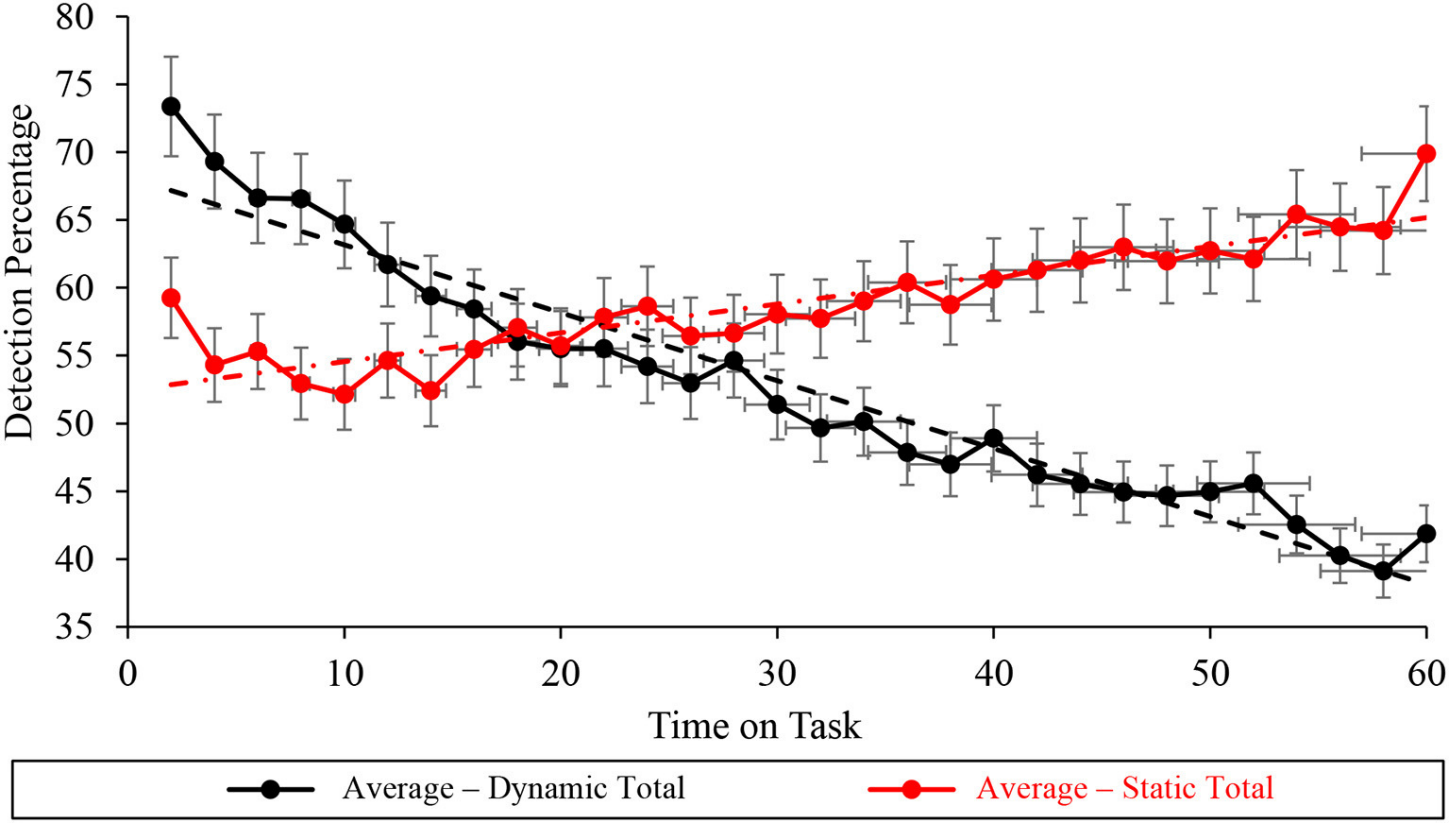
Graph displaying the average detection percentage recorded at the total task level for all the static (red) and dynamic (black) WACDT trials, with horizontal and vertical error bars indicating the standard deviation.

The Durbin-Watson analysis undertaken at the total task level of the average correct detection percentage data suggested statistically significant autocorrelation under dynamic (*D* = 0.516 < *d*_*u*_ = 1.489) and static (*D* = 1.178 < *d*_*u*_ = 1.489) conditions (Table 2, Figure 8). Mann-Kendall Sen's Slope estimates of WACDT vigilance performance trajectories at the total task level demonstrated statistically significant improvement and decrement during static (*S* = 0.440, *p* < 0.001) and dynamic (*S* = −0.948, *p* < 0.001) WACDT conditions respectively. Moreover, Mann-Kendall Sen's Slope estimates of total task vigilance performance visually aligned with the positive and negative trajectories respectively observed in the total vigilance performance plots under static and dynamic conditions (Figure 8).

**Table 2:** Vigilance performance trend analysis.

**Assumption tests and trend analyses**	**Dynamic across all screens**	**Static across all screens**	**Dynamic left**	**Static left**	**Dynamic center**	**Static center**	**Dynamic right**	**Static right**
Durbin-Watson D Statistic	0.516	1.178	1.553	1.053	1.507	0.917	1.108	1.850
D-lower	1.352	1.352	1.352	1.352	1.352	1.352	1.352	1.352
D-upper	1.489	1.489	1.489	1.489	1.489	1.489	1.489	1.489
Significant autocorrelation	yes	yes	no	yes	no	yes	yes	no
Mann-Kendall MK statistic	−407	339	−195	219	−167	181	97.000	303
Standard Error	56.051	56.051	56.051	56.051	56.051	56.051	56.051	56.051
Z-stat	−7.243	6.030	−3.461	3.889	−2.962	3.211	1.713	5.388
*P*-value	<0.01	<0.01	<0.01	<0.01	<0.01	<0.01	<0.05	<0.01
Trend	Negative	Positive	Negative	Positive	Negative	Positive	Positive	Positive
Sens slope s statistic	−0.948	0.440	−0.729	0.233	−1.770	0.512	0.284	0.211
S-lower	−1.080	0.368	−1.042	0.149	−2.591	0.262	−0.041	0.158
S-upper	−0.863	0.517	−0.423	0.341	−0.612	0.807	0.542	0.277

### Left screen subtask vigilance performance

**Figure 9:**
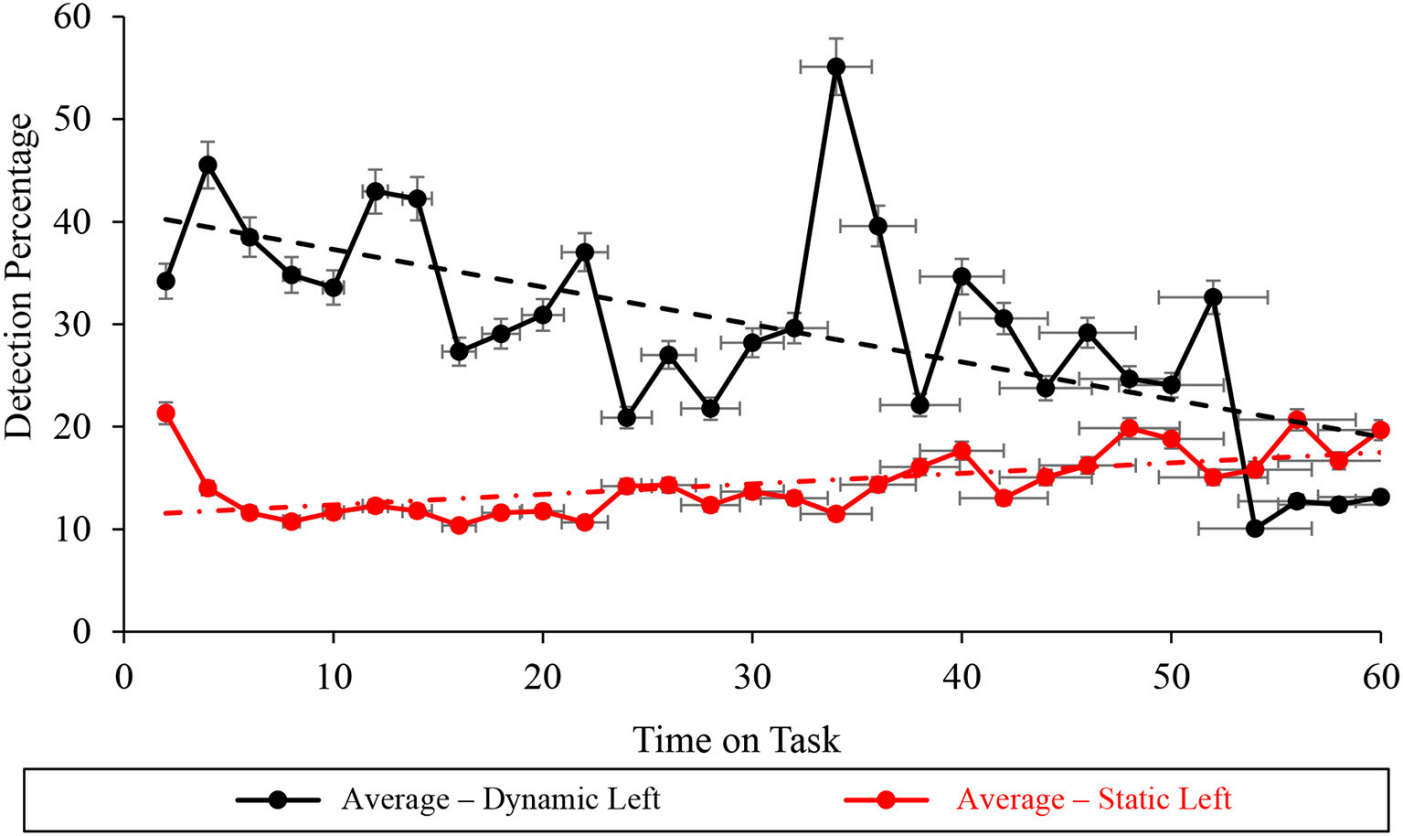
Graph displaying the average detection percentage recorded at the left screen level for all the static (red) and dynamic (black) WACDT trials, with horizontal and vertical error bars indicating the standard deviation.

Autocorrelation in the left subtask level of the averaged correct detection percentage vigilance performance was statistically non-significant during dynamic (*D* = 1.533 > *d*_*u*_ = 1.489) conditions and significant during static (*D* = 1.053 <*d*_*u*_ = 1.489) WACDT conditions (Table 2, Figure 9). Mann-Kendall Sen's Slope estimates of vigilance performance on the left screen subtask respectively demonstrated statistically significant improvements and decrements with time on task, during static (*S* = 0.233, *p* < 0.001) and dynamic (*S* = −0.792, *p* < 0.001) WACDT conditions (Table 2, Figure 9). Each follow-up visual analysis of vigilance performance on the left screen subtask during static and dynamic WACDT conditions, respectively, demonstrated positive and negative trajectories, which affirmed Mann-Kendall Sen's Slope trend estimate (Figure 9).

### Center screen subtask vigilance performance

**Figure 10:**
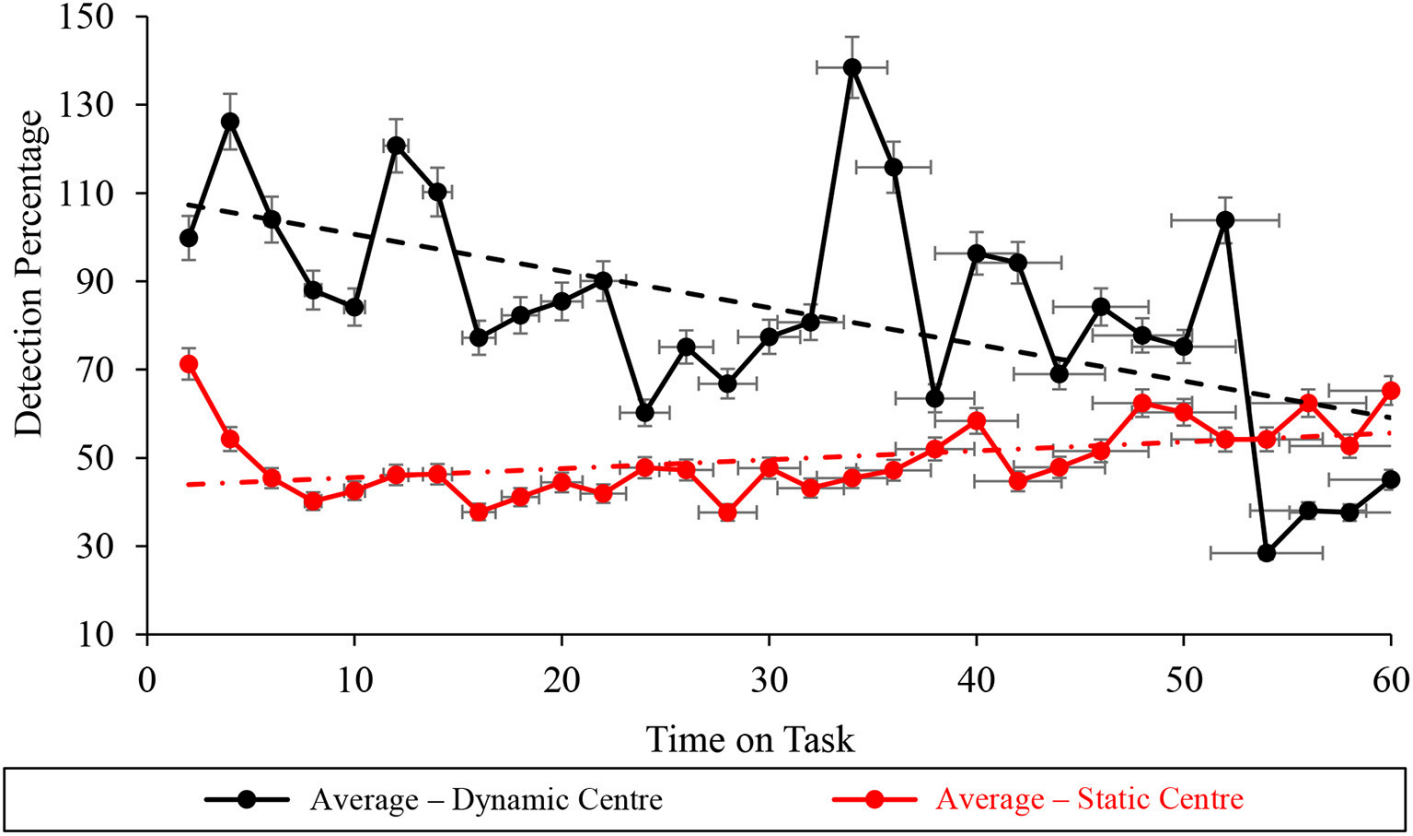
Graph displaying the average detection percentage recorded at the center screen level for all the static (red) and dynamic (black) WACDT trials, with horizontal and vertical error bars indicating the standard deviation.

Durbin-Watson analyses of the vigilance performance on the WACDT's center subtask demonstrated statistically significant autocorrelation under static (*D* = 0.917 <*d*_*u*_ = 1.489) conditions (Table 2, Figure 10). In contrast, the Durbin-Watson analysis of vigilance performance data recorded during dynamic WACDT conditions demonstrated non-significant autocorrelation (*D* = 1.507 > *d*_*u*_ = 1.489). Statistically significant improvements and decrements were then demonstrated by Mann-Kendall Sen's Slope estimates of vigilance performance trajectory on the center subtask measured during static (*S* = 0.512, *p* = 0.001) and dynamic (*S* = −1.770, *p* = 0.002) WACDT conditions. Downward trends were observed from each center screen performance plot, which was corroborated by the trends estimated by Mann-Kendall Sen's Slope analyses (Figure 10).

### Right screen subtask vigilance performance

**Figure 11:**
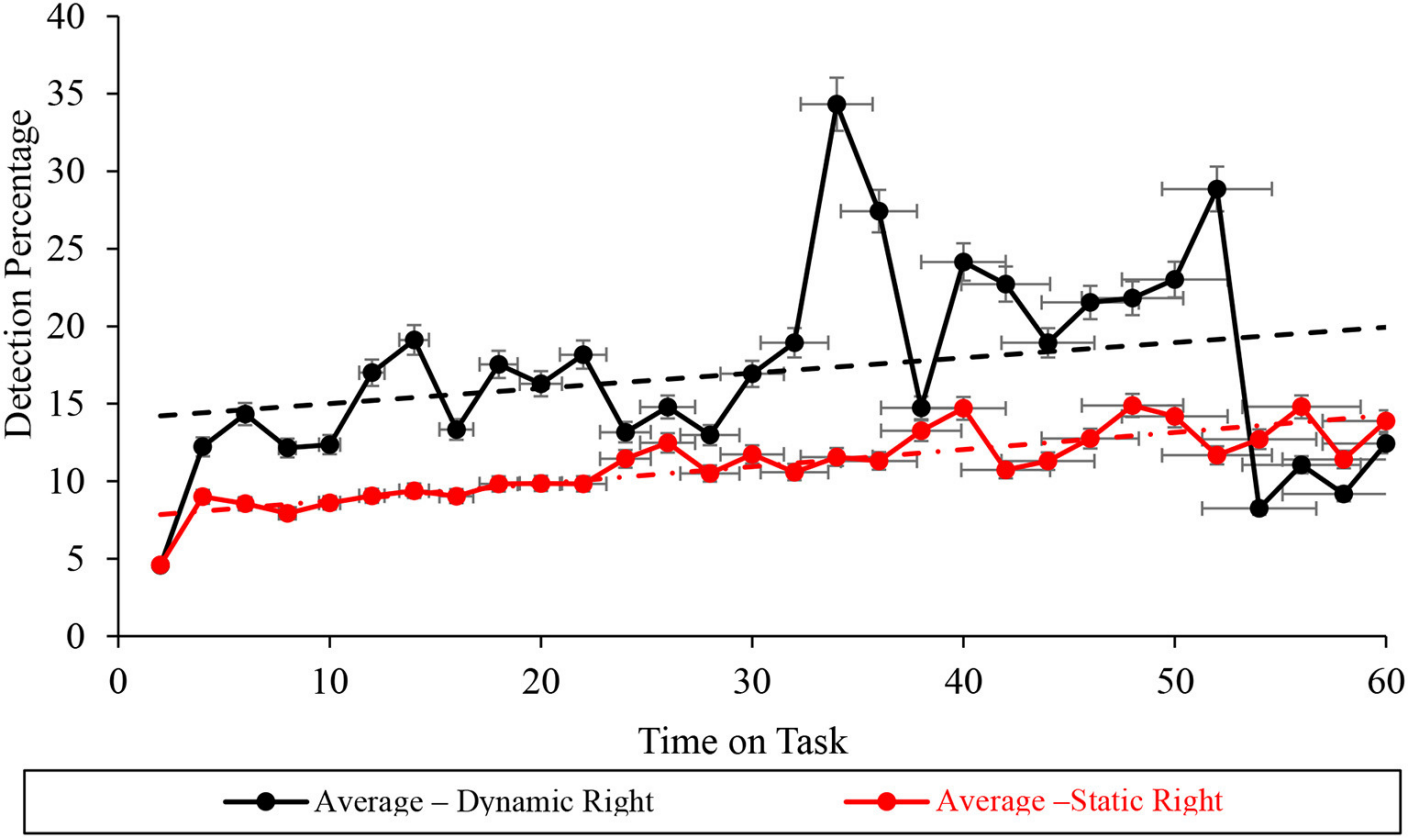
Graph displaying the average detection percentage recorded at the right screen level for all the static (red) and dynamic (black) WACDT trials, with horizontal and vertical error bars indicating the standard deviation.

Durbin-Watson analyses demonstrated statistically significant autocorrelation within the right screen subtask vigilance performance data, recorded during dynamic (*D* = 1.108 <*d*_*u*_ = 1.489), but not static (*D* = 1.805 > *d*_*u*_ = 1.489) WACDT conditions (Table 2, Figure 11). Furthermore, Mann-Kendall Sen's Slope estimates of the vigilance performance trajectory during the right screen subtask respectively demonstrated statistically significant improvements during both static (*S* = 0.211, *p* < 0.001) and dynamic (*S* = 0.284, *p* = 0.043) conditions of the WACDT. Moreover, each trend estimated by Mann-Kendall Sen's Slope analyses, undertaken over the right screen subtask vigilance performance data, aligned with the positive trajectories visually observed in graphs of both static and dynamic conditions (Figure 11). Each vigilance performance curve has been plotted with standard error bars.

### Vigilance performance compared between the group average and individuals

Psychological phenomena cannot be assumed to manifest the same way in individuals as in ensemble sample averages because the human brain is a non-ergodic system (Speelman and McGann, 2020). Comparing the sample- with individual-level analyses of vigilance performance on the WACDT, however, facilitated a check of the ergodicity within the data (Speelman and McGann, 2020).

Vigilance performance on the dynamic WACDT was first examined at the total-task level, T(t). Sample level analyses of the dynamic WACDT's data set, D_avg_, demonstrated a decrement in vigilance performance with time on task. At the individual level, 24 out of 25 participants demonstrated decrements in vigilance performance with time on the dynamic WACDT. Only four percent of participants deviated from the gradual decrement in vigilance performance with time spent on the dynamic WACDT, as demonstrated at the sample level. The remaining 96 percent of participants demonstrated vigilance performance curves aligned with the sample average, namely a gradual decrement over time. In contrast, sample-level analyses of the static WACDT data set, $\overset{\bar{}}{S}$, demonstrated an improvement in vigilance performance with time on task that aligned with ninety-six percent of participants' individual-level analyses. Only one participant demonstrated a decrement in vigilance performance during the static WACDT.

Table 3 summarizes the conclusions drawn across all 26 data sets analyzed by visual inspection of vigilance performance curves and Sen's Slope and Mann-Kendall Analyses.

**Table 3:** Comparison of conclusions derived by visual and analytic exploration of vigilance performance trajectories.

**Comparison**	**Trial**	**Set count**	**Prevalence**
Analytically reported improvement aligns with visually observed improvement	Static	25	96.15%
	Dynamic	0	0.00%
Analytically reported decrement aligns with visually observed decrement	Static	1	3.85%
	Dynamic	22	84.62%
Analytically reported improvement misaligned with visually observed decrement	Static	0	0.00%
	Dynamic	1	3.85%
Analytically reported improvement misaligned with visually observed improvement	Static	0	0.00%
	Dynamic	3	11.54%

A total of 52 comparisons were undertaken between vigilance performance trajectories, including the group average data. Only four out of twenty-five instances in which the vigilance performance trajectory derived by the Sen's Slope and Mann-Kendall Analyses did not match the trends visually observed in the data plots. Namely, this included participants 1, 12, 15, and 16 during their dynamic trials. However, the trends visually observed in plots of the data aligned with the vigilance performance trajectory derived by the Sen's Slope and Mann-Kendall Analyses for 96.15% of static trials where performance was seen to improve, and 84.62% of dynamic trials where performance declined over time.

Furthermore, vigilance performance improved in 96.15% of static trials and declined in 84.62% of dynamic trials, which can only be attributed to the different ways cognitive load, signal salience, and event rate were operationalised between the two conditions. This supports the notion that Parasuraman (1979, 1985) parameters are generalisable task performance factors that influence vigilance performance in traditional as well as novel modern paradigms (Grier et al., 2003; Oken et al., 2006; McIntire et al., 2011, 2013; Knott et al., 2013; Neigel et al., 2020).

### The reliability, validity, and longevity of the WACDT

The reliability of the WACDT was assessed under both static and dynamic conditions. Two split-half reliability coefficients were derived by first calculating the minute-by-minute detection percentages for both static and dynamic WACDT trials. This resulted in two distinct data sets, each containing 60 values. These values represent the average detection percentages for each minute under static and dynamic conditions, respectively. Next, each 60-value set were sorted into two groups based on the timestamp: one with odd-numbered timestamps and another with even-numbered timestamps. The 30 odd-timestamped values were then correlated with the 30 even-timestamped values within each condition; static and dynamic. This process yielded the two split-half reliability coefficients for the WACDT under both static and dynamic conditions. The split half reliability coefficient for the dynamic condition was r_(60)_ = 0.959, *p* < 0.001 and r_(60)_ = 0.848, *p* < 0.01 under static conditions. Both the dynamic and static trial split-half coefficients were above the critical value of 0.75 that Portney and Watkins (2015) suggested high reliability. The WACDT was thus demonstrated to be a reliable cyber vigilance task.

Guidetti et al. (2023) review identified longevity as a limitation of existent vigilance tasks vigilance presented by McIntire et al. (2013), Mancuso et al. (2015), and Sawyer et al. (2016). For example, as the tools, technologies and methods that cyber vigilance tasks aim to emulate evolve rapidly (Guidetti et al., 2023). This begets the need to regularly update cyber vigilance tasks, so that they continue to accurately emulate the demands associated with network defense. This is not dissimilar to psychological instruments like the Wechsler Adult Intelligence Scale, which also require routine updates to maintain validity. For example, the tasks McIntire et al. (2013), Mancuso et al. (2015), and Sawyer et al. (2016) presented may have served the purposes of a cyber vigilance task well at their inception. However, their validity by today's standards is unclear, as cyber defense has evolved beyond tools of their level of complexity. Though it is difficult to predict with any precision, when the WACDT's longevity might come into question, as that depends on the rate that technology changes in network defense. Providing regular updates and improvements to the WACDT therefore reflects an essential effort required to maintain its longevity and validity and avoid rapid obsolescence.

As well as avoiding obsolescence, providing additional periodic updates to the WACDT could enhance its ecological validity as a cyber vigilance task. For example, a cognitive task analysis (CTA) of SEIM consoles surveyed by industry members could be undertaken every six to eight years and used to inform new features to include in future iterations of the WACDT. This could include expanding the number of screens to present the WACDT or adding more subtasks developed in network command-and-control consoles.

## Discussion

The primary aim of this study was to present the WACDT as a new, novel, accessible and validated cyber vigilance task. It was hypothesized that the trajectory of WACDT performance would decline with time on task. Two versions of the WACDT were tested. Under dynamic conditions, each parameter increased in difficulty with time on task. In contrast, each parameter was set to the most challenging level of processing. Thus, it was also hypothesized that differences in signal salience, event rate and cognitive load implemented in the static and dynamic forms of the WACDT would influence the trend component of WACDT performance declines.

Sen's Slope and Mann-Kendall Analyses were used to derive trends for the total, T(t), WACDT vigilance performance observed across each participant and the group average. These analytically derived trends were then compared to trajectories derived by visually observing plots for each data set. Across static and dynamic trials of the WACDT, this comprises 52 sets of vigilance performance data. That is, 25 data sets were recorded from dynamic trials, 25 data sets from static trials, and an averaged data set was computed for each, for a total of 52. In total 96.15% of static WACDT trends derived by visual inspection and Sen's Slope and Mann-Kendall Analyses demonstrated improved vigilance performance.

In contrast, vigilance decrement was observed in 84.62% of dynamic WACDT trends derived from visual inspection of the data and Sen's Slope and Mann-Kendall Analyses. Of the 52 comparisons of WACDT vigilance performance, only three instances in which the trajectories calculated by Sen's Slope and Mann-Kendall Analyses did not match what was visually observed in data plots. That is, vigilance performance improved in most static WACDT data sets. Likewise, vigilance performance declined in a majority of dynamic WACDT data sets.

Vigilance performance on the static and dynamic WACDT versions was explored at the level of total task performance T(t), as well as within the left, L(t), center, C(t), and right R(t) screen subtasks. The decline in vigilance performance observed during the dynamic WACDT supported the first hypothesis. Namely, if the WACDT is a valid vigilance task, then performance will decline over time. Vigilance performance on the static form of the WACDT improved, in contrast to the decline observed during the dynamic version of the task (Table 2, Figure 8). This result was also seen at the WACDT's subtask level, L(t), C(t), and R(t), with only one exception, namely, vigilance performance on the right screen subtask, R(t), improved under both the dynamic and static WACDT conditions (Figures 6–9).

The decrements in vigilance performance observed during the dynamic WACDT and improvements observed during the static version of the task demonstrated support for the second hypothesis. Namely, if the WACDT is a valid vigilance task, then dynamically increasing signal salience, event rate and cognitive load during the dynamic WACDT should lead to greater performance deficits than in the static version where each parameter was kept constant (Parasuraman, 1979, 1985). Vigilance performance improved across each static condition subtask, whereas a decrement was observed on all subtasks bar the dynamic condition right screen subtask, R(t). The improvement in vigilance performance observed on the dynamic right screen subtask could be explained by the close resemblance between this WACDT component and meta-data anomaly detection, a job that network defense analysts perform in real-world cyber security operations centers (Keyvanpour et al., 2020). However, this could indicate that vigilance performance on the WACDT was best captured at the total task level rather than by any one subtask. The Western Australian Cyber Defense Task was therefore supported as a valid vigilance task since vigilance decrement was observed in the dynamic form of the WACDT, whereas improvement was observed in the static version.

### Relationship between current and prior research

The United States Wright Patterson Airforce Research Lab possesses the only existing cyber vigilance tasks with which the WACDT could be compared (Guidetti et al., 2023). In contrast to these existent tasks, the WACDT is a validated and accessible cyber vigilance task that can be accessed by parties external to the United States Wright Patterson Airforce Research Lab.

### Implications

The WACDT can serve as an experimental testbed for human factor cyber security research. Beyond research, however, the WACDT holds implications for how cyber security command-and-control consoles are built and maintained. On a practical level, cyber security software engineers could use the psychophysical boundaries of signal salience, event rate, and cognitive load in the WACDT to tailor cyber command-and-control systems to suit analysts' neuro-cognitive capabilities. For example, suppose a software engineer designs, develops, and deploys a commercial SEIM within a cyber security operation center. The WACDT could calibrate a neuro-ergonomic composition of signal salience, event rate, and cognitive load that minimizes analysts' vigilance decrement performance. An analyst could first perform a version of the WACDT calibrated to match the signal salience, event rate, and cognitive load of the company's command-and-control console. The analyst could then perform a range of WACDT trials under a range of signal salience, event rate, and cognitive load compositions to identify one that optimizes their individual sustained attention capacity. This procedure could also benchmark the sustained attention capacity of new hire analysts without exposing candidates to core details about a company's command-and-control console.

### Limitations and future research

Several factors limit the extent to which the WACDT might be generalized beyond the laboratory to the wider population of network defense analysts. These include sample size, the range of dynamic WACDT parameters tested, and task duration. Given that the sample of analysts who participated in this research reasonably approximated the age and gender distribution and years of operational experience of the wider Australian cyber security analyst population, it may generalize well at the moment (ISC^2^, 2020). However, as the gender distribution within the wider cyber security changes over time, the generalisability of this work may decrease over time. Therefore, moving forward, an avenue of future research would be to test the WACDT on a larger, more diverse sample of network defense analysts.

The population average age of cyber security professionals is 42 years old; however, the sample had an average age of M_age_ = 35.68 years old with σ_age_ = 11.93 years (ISC^2^, 2020). Given that the sample average was younger than the population average, the conclusions derived through this work may not generalize to older members of the population of network security analysts. An avenue of future research would also be to explore the impact of age on cyber vigilance performance. For instance, age is associated with increased vigilance decrement; however, Parasuraman and Giambra (1991) also demonstrated that experience can moderate this relationship.

Secondly, because the sample comprised operational network defense analysts, their employment responsibilities limited the amount of time they could reasonably dedicate to completing WACDT trials. The static and dynamic versions of the WACDT were designed to explore the sensitivity of vigilance performance to changes in signal salience, event rate and cognitive load. Each parameter was made to fluctuate simultaneously within the dynamic task. However, there are multiple ways that sensitivity to each parameter could have been explored. However, this would have also increased the time commitment required from each participant to complete the research from approximately 2 to 8 h per participant. In the future, different compositions of dynamic parameter variation may inform features of network defense, which differentially impact cyber vigilance performance. For example, an alternative form of dynamic trial could have the event rate set as fixed while signal salience and cognitive load vary. This form of dynamic trial could facilitate an exploration of the interaction between the cognitive load associated with processing a SEIM alert and recognizing its threat level. What is the relationship between the complexity of a cyber-attack and its obviousness as a problem to an operator?

Related to the second limitation was the 60-min time limit of each WACDT trial. Most laboratory vigilance tasks are run for 40 min to an hour (See et al., 1995; Helton et al., 1999; Warm et al., 2008, 2009; See, 2014). However, Chappelle et al. (2013) reported that network defense analysts often work up to 10.5 h per day, with minimal rest periods, for a total of 52.5 h per week. The demands associated with 60 min of the WACDT cannot compare to the 10.5 h per day that Chappelle et al. (2013) observed as the root cause of clinically significant burnout and stress in network defense analysts (O'Connell, 2012; Mancuso et al., 2015). Operational limitations on participants' time prevented testing WACDT performance for periods longer than an hour. The WACDT's external validity was limited by the hour-long constraint imposed on task duration. Future studies, however, should explore cyber vigilance performance over time periods that more closely approximate what is required in the real world.

Under dynamic WACDT conditions, there was a decline in vigilance performance for the left and center screen subtasks. However, there was an improvement in vigilance performance for the right screen subtasks. The reason for this divergent trend remains unclear. One possibility is that participants were more accustomed to anomaly detection tasks, like those simulated on the right screen. Their prior experience in operational SEIMs could have influenced their performance in the WACDT environment. Further research is needed to fully understand the underlying factors for this discrepancy in performance across the different screens.

## Conclusion

In closing, the WACDT is the most up-to-date cyber vigilance task that civilian human factors researchers can use to study declines in sustained attention during network defense. Unlike existent cyber vigilance tasks, the WACDT was designed with the ability to control each of the parameters that Parasuraman (1979, 1985) suggested influenced declines in sustained attention. Human factors researchers could leverage the WACDT to study ways of managing the risk associated with vigilance decrement in operational network defense. For example, this could include understanding how different compositions of signal salience, event rate, cognitive load, and workload transitions influence the cyber defensive capacity of network defense analysts working with cyber command-and-control consoles.

## Author's note

In following with our recently published article, “A Review of Cyber Vigilance Tasks for Network Defense” we now wish to submit another original research article, entitled “The Western Australian Cyber Defense Task” for publication consideration. We confirm that this work is original and has not been published elsewhere, nor is it currently under consideration for publication elsewhere. In this paper, we present a new vigilance task for cyber security, that overcomes the challenges outlined in our previous work and which can be used to assess the capacity of network defense analysts to sustain attention to virtual threats presented in modern Security Event Information Management software. This is significant because we overcome the challenges associated with developing a modern and updated cyber vigilance task outlined in our previous publication. We believe the aims and scope of Frontiers in Neuroergonomics is ideal for our manuscript which address a range of interdisciplinary challenges that limit the study of symbiotic human computer interactions in the cyber security domain. Moreover our task fills an important gap in the literature, namely a tool that researchers can use to study the impact of neuroergonomic features of cyber security command and control console software.

## Data availability statement

The raw data supporting the conclusions of this article will be made available by the authors, without undue reservation.

## Ethics statement

The studies involving humans were approved by Edith Cowan University Human Research Ethics Committee. The studies were conducted in accordance with the local legislation and institutional requirements. The participants provided their written informed consent to participate in this study.

## Author contributions

All authors listed have made a substantial, direct, and intellectual contribution to the work and approved it for publication.
